# Deep Vein Thrombosis Following Below Knee Immobilization: The Need for Chemoprophylaxis

**DOI:** 10.5812/traumamon.9158

**Published:** 2013-01-15

**Authors:** Alireza Manafi Rasi, Gholamhossein Kazemian, Mohammad Emami Moghadam, Reza Tavakoli Larestani, Amirhossein Fallahi, Ali Nemati, Maryam Nazari, Fateme Fallahi, Saeed Safari

**Affiliations:** 1 Department of Orthopedic and Trauma Surgery, Shahid Beheshti University of Medical Sciences, Imam Hossein Hospital, Tehran, IR Iran; 2 Department of Physical Medicine and Rehabilitation, Tehran University of Medical Sciences, Firuzgar Hospital, Tehran, IR Iran; 3 Kashan Nursing and Midwifery College, Kashan University of Medical Sciences, Kashan, IR Iran; 4 Department of Emergency Medicine, Shahid Beheshti University of Medical Sciences, Imam Hossein Hospital, Tehran, IR Iran

**Keywords:** Venous Thrombosis, Immobilization, Knee Joint, Prophylaxis, Venous Thrombosis, Immobilization, Knee Joint, Prophylaxis

## Abstract

**Background:**

There is controversy regarding routine prophylaxis for deep vein thrombosis (DVT) in patients treated via a short leg cast or splint following lower extremity trauma.

**Objectives:**

The main aim of this study is to evaluate the incidence of DVT and need for chemoprophylaxis in these patients.

**Materials and Methods:**

Patients with ankle sprains or stable foot/ankle fractures were entered in this cross-sectional study. Serum D-dimer levels were measured 2 weeks following fixation. If the D-dimer levels were above 0.2 micrograms/ml the test was considered positive and the patient was referred for Doppler ultrasound examination (DUE) to confirm or rule out the diagnosis of DVT. Finally, the incidence of DVT was calculated and the role of predisposing factors was investigated.

**Results:**

There were 95 patients with an average age of 38 ± 13.7 (77.9% males); 46 patients had at least one risk factor for DVT. The D-dimer test was positive in 21(22.1%) patients. DVT was confirmed by DUE in 3 patients (3.1%). The incidence of DVT significantly increased in the presence of 3 or more risk factors (P = 0.01).

**Conclusions:**

It seems that DVT is not a common complication of below knee fixation and chemoprophylaxis is not necessary when the patient has less than 3 predisposing factors. With 3 or more risk factors chemoprophylaxis and periodic follow-ups must be considered.

## 1. Introduction

Diagnosis of DVT is commonly made for many emergency department visits and numerous studies have been conducted to evaluate its predisposing factors. Immobilization is one of the main predisposing factors of DVT (1). Currently many patients with stable foot/ankle fractures or ligament injuries are treated a splint or a short leg cast, which is said to predispose the affected leg to DVT due to immobilization and inactivity of the ankle pump mechanism (2). Previous studies have reported the incidence of DVT after non-surgical treatment of lower extremity injuries to be between 1.1 and 20% (2). Nonetheless, the true incidence of DVT and the need for prophylaxis is not yet clear (3). Worldwide, many centers have started using various methods of chemoprophylaxis for these patients routinely (4). Considering the large volume of patients who seek medical attention with these types of injuries, routine use of DVT chemoprophylaxis would put an unbearable financial burden on the health systems. The aim of the present study is to evaluate the incidence of DVT and the need for chemoprophylaxis in this group of patients.

## 2. Material and Methods

A prospective cross-sectional study was designed. Patient consent was obtained and the patients were recruited from the emergency department and orthopedic clinic where they were first visited and evaluated with complaints of foot and ankle pain. If the diagnosis of a stable fracture or ligament injury of the foot or ankle was made, a short leg splint or cast was advocated for 4-6 weeks. Inclusion criteria for selection were patients with stable foot or ankle fractures (undisplaced or minimally displaced fractures) or grade 3 lateral sprained ankle without instability and 15 years age or over. The fractures included lateral malleolus fractures with intact medial side and intact tibiofibular syndesmosis and hind/mid/forefoot fractures fulfilling radiographic criteria for non-surgical treatment. Patients younger than 15 y/o age and those that had instability of ankle (medial side of ankle or syndesmosis injuries) or foot (displaced fractures or multiple injuries of foot) were excluded. A circular cast was applied from below the fibular head to the metatarsophalangeal joints. Between days 7 and 14, when the risk of DVT is said to be at its highest, 5 ml of blood was drawn for D-dimer level measurement by ELISA. In the patients with a positive test (D-dimer > 0.2micrograms/ml), the diagnosis of DVT was ruled in/out by Doppler ultrasound, performed by two different radiologists independently. In this study age over or equal to 40, female gender, body mass index (BMI) above 30 kg/m2, history of cardiovascular/cerebrovascular accidents (Chronic heart failure, myocardial infarction), multiple trauma, smoking, use of oral contraceptives, history of malignancy, previous history of DVT, varicose veins and immobilization for more than 3 days were considered risk factors for venous thrombosis. The patients were forewarned about the signs and symptoms of DVT at the time of treatment and were followed closely to the end of their treatment period. The relationship between the risk factors and the incidence of DVT was tested with chi-square and Fisher’s exact tests and the significance level was set at 0.05.

## 3. Results

**Figure 1:**
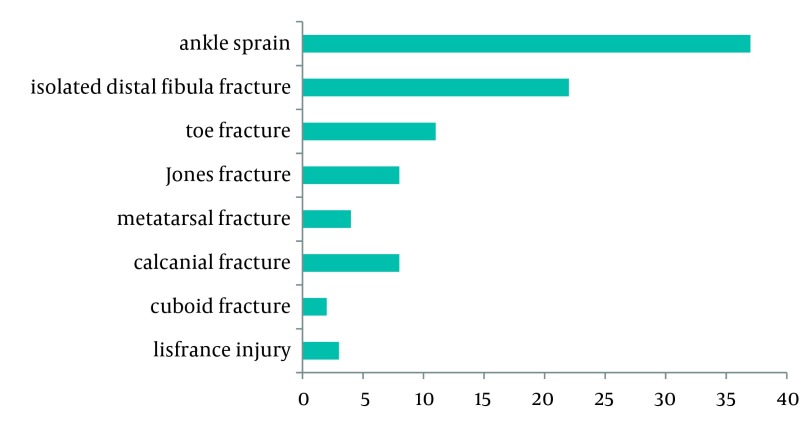
Distribution of Patients According to the Type of Injury

A total of 95 patients with ligament injuries or stable fractures of the foot or ankle were studied (77.9% males). The average age was 38 ± 13.7 (range 15-71) and 32 patients were older than 40. Figure 1 shows the distribution of study subjects according to the type of injury.

**Figure 2:**
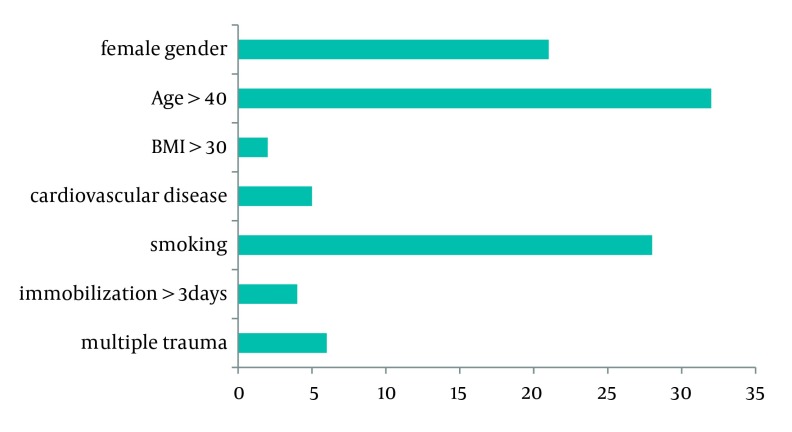
Distribution of Risk Factors in the Study Patients

The average BMI was 25 ± 3.4 kg/m2 (range 18.4-38.5) and in two patients the BMI was over 30. Five patients (5.3%) had history of cardiovascular or cerebrovascular disease and 28 patients (29.5%) were smokers. Four patients were immobilized for more than 3 days and 6 patients had multiple trauma. A total 46 patients had at least one risk factor for DVT (24 patients had one risk factor, 16 patients two risk factors and 6 patients 3 risk factors). Figure 2 shows the distribution of risk factors in the study patients;

21 (22.1%) patients had positive D-dimer levels. Ultrasound confirmed DVT in only 3 out of the 21 patients (3.1% of total patients) and clinical signs and symptoms were present in only 1 patient. These 3 patients had an average D-dimer level of 2.05 ± 0.74. The three DVT patients included a 65-year-old lady with an ankle sprain who had been immobilized for a week at home. She came back with signs of popliteal venous thrombosis. The second patient was a male smoker aged 52 years with an isolated lateral malleolus fracture who had sustained multiple trauma. He had posterior tibial venous thrombosis. The third patient was a 36-year-old man with calcaneal fracture who developed peroneal DVT. We hospitalized these 3 patients. According to our hospital protocol because of inaccessibility to a vascular surgeon, we consulted with internal medicine specialist and started therapeutic dosage of Enoxeparine and if needed the patient was referred to vascular surgeon in another hospital. The patients cast was removed and treatment with splint continued up to completion of treatment.

There was no significant difference between patients with one or two DVT risk factors and the rest of the patients with regards to the incidence of venous thrombosis. However, when three or more risk factors existed the difference became significant (P = 0.01). In our study 33% of patients with multiple risk factors (three or more) developed DVT.

## 4. Discussion

According to the results of this study, immobilization alone is not a strong enough predisposing factor to affect the incidence of DVT in patients with a short leg splint or cast per se. However, the risk of DVT increases significantly in patients with 3 or more predisposing factors. DVT is a common problem after trauma to foot and ankle and has always been challenging to diagnose and treat (5, 6). Although the incidence of DVT after short leg casting is reported between 1.1-20% (7, 8), its occurrence after ankle sprains or isolated stable foot/ankle fractures treated in a short leg cast and indications for pharmacologic thromboprophylaxis remain unknown (9). The American College of Chest Surgeons discourages routine use of thromboprophylaxis for isolated lower extremity fractures (10). In this study DVT was confirmed sonographically only in three (3.1% of total) patients and was symptomatic in only one patient. In the study on incidence of venous thrombosis in patients with isolated ankle fractures, treated in short leg casts, Patli and colleagues studied 100 patients without any prophylaxis. After removal of the cast they noted 5 cases of asymptomatic DVT diagnosed by means of ultrasound (5). Giannadakis et al., stated that the incidence of DVT in minor lower extremity injuries was about 1.1% and that it did not need any prophylaxis (7). On the other hand some investigators believe that DVT after lower extremity casting is an important problem with an incidence of 20% and some form of prophylaxis is necessary (8). In our study no relationship was found between DVT and any one particular risk factor, however, the incidence of DVT was significantly higher in patients with three or more risk factors. Therefore, it seems necessary to consider patients with multiple risk factors for thromboprophylaxis or warn the patient at the time of treatment about the signs and symptoms of DVT and ask them to return to the clinic immediately if any of those signs and symptoms develop.

It seems that DVT is not a common complication of below knee fixation and chemoprophylaxis is not necessary when the patient has less than 3 predisposing factors. With 3 or more risk factors chemoprophylaxis and periodic follow-ups must be considered.
